# Development of Flavor and Taste Components of Sous-Vide-Cooked Nile Tilapia (*Oreochromis niloticus*) Fillet as Affected by Various Conditions

**DOI:** 10.3390/foods11223681

**Published:** 2022-11-17

**Authors:** Jaksuma Pongsetkul, Jirawat Yongsawatdigul, Surintorn Boonanuntanasarn, Soottawat Benjakul

**Affiliations:** 1 School of Animal Technology and Innovation, Institute of Agricultural Technology, Suranaree University of Technology, Nakhon Ratchasima 30000, Thailand; 2 School of Food Technology, Institute of Agricultural Technology, Suranaree University of Technology, Nakhon Ratchasima 30000, Thailand; 3 International Center of Excellence in Seafood Science and Innovation, Faculty of Agro-Industry, Prince of Songkla University, Songkhla 90110, Thailand

**Keywords:** sous-vide, volatile compounds, taste/flavor components, Nile tilapia, principal component analysis

## Abstract

This study aims to shed light on the association between non-volatile and volatile compounds related to flavor/taste characteristics as well as sensory acceptability of Nile tilapia fillet (*Oreochromis niloticus*) cooked by various sous-vide (SV) conditions (50–60 ℃, 30–60 min), with fish cooked with boiling water used as control. Higher temperatures and longer processing times of SV cooking led to greater protein and lipid oxidation as indicated by the increase in total sulfhydryl (-SH), carbonyl, free fatty acid (FFA) contents as well as peroxide values (PV) and thiobarbituric acid reactive substance (TBARS) values. The differences in flavor/taste components including adenosine triphosphate (ATP)-related compounds, free amino acids (FAAs) and volatiles were also obtained, which directly affect sensory acceptability as evaluated by using the hedonic scale. Based on principal component analysis (PCA) results, the acceptability score was strongly correlated with inosine monophosphate (IMP) and acetoin, which seem to be the most crucial flavor enhancers for cooked tilapia. Among all samples, tilapia processed at 60 °C for 45 and 60 min, which contained significantly higher IMP and acetoin (*p* < 0.05) than others, had significantly higher flavor-liking and overall-liking scores, with a more than 7.5 meaning for high acceptability (*p* < 0.05), indicating the optimal SV conditions for tilapia fillet. Overall, the present finding indicated that the SV-cooking technique, at the optimal conditions, can improve the meat quality of cooked fish, in terms of flavor/taste characteristics, compared with traditional cooking (control).

## 1. Introduction

Sous vide (SV), which means “under vacuum” in French, is an emerging cooking technique, which includes vacuum-sealing food in a bag, then cooking it to a very precise temperature in a water bath. Compared to traditional cooked, SV cooking uses a lower temperature (ranging from 50–85 °C) and a prolonged time of up to 12–24 h depending on each type of meat [1]. This technique has become popular and widely used in many food enterprises over the last decade. For fish meat, the benefits of the SV method have been reported in several articles that studied various fish species, including salmon (*Salmon salar*) [2], tuna (*Thunnus maccoyii*) [3], trout (*Oncorhynchus mykiss*) [4] or Nile tilapia (*Oreochromis niloticus*) [5], etc. It was noted that SV-cooked fish had better color, texture and nutrient retention. The shelf-life of SV products is also improved since this cooking technique can eliminate the risk of recontamination, inhibit off-flavors from oxidation as well as prevent some volatile and moisture losses during cooking and storage.

Flavor/taste are important characteristics determining a product’s quality. In general, different cooking temperatures and times can modify the volatile profiles and consequently the cooked meat’s odor/flavor [6]. Most volatile compounds in cooked meat are generated due to the thermal degradation of lipids and protein. Lipid degradation, both lipolysis and oxidation, has been commonly associated with the development of flavor and aroma [7]. At any rate, these changes contribute to the formation of the typical and desirable odor/flavor of cooked meat, whereas long-term cooking resulted in serious lipid oxidation and protein oxidation, which may cause an undesirable flavor instead. During cooking, continuous high temperatures can increase the generation of reactive oxygen species (ROS), i.e., free radicals and non-radicals, which can strengthen the tendency of protein and lipid oxidation [8]. Non-volatile compounds are also responsible for contributing to meat’s taste. Nucleotides, such as inosine-5′-monophosphate (IMP), and amino acids, such as glutamic acid, play a significant role in providing umami taste [9]. Pegg and Shahidi [10] stated that the taste/flavor of cooked meat is mainly developed upon heating treatment, with more than 100 volatiles/non-volatiles compounds described. However, only some of them play a significant role in the overall flavor characteristics of cooked meat. Thus, cooking conditions play a crucial role for the development of desirable flavor/taste to the product. To date, the taste/flavor characteristics of SV-cooked fish have yet to be explored, particularly both volatiles and non-volatiles, as well as their relationship with consumer preference and acceptance. Therefore, this study aimed to investigate the taste/flavor information of SV-cooked tilapia as affected by various SV conditions, concerning both temperature and time. Nile tilapia (*O. niloticus*) was selected since it is one of the most important aquaculture species, and is highly appreciated and consumed in the world, particularly in Asia. Furthermore, the relationship between taste/flavor compounds and the sensory acceptability of SV-cooked tilapia processed by various SV conditions was also studied using principal component analysis (PCA). With these approaches, an insight into the understanding of desirable flavor/taste developments of SV-cooked fish that directly affect product quality will be achieved.

## 2. Materials and Methods

### 2.1. Sample Preparation

Nile tilapia (*O. niloticus*) weighing 656.25 ± 82.26 g were obtained from Suranaree University of Technology farm (Nakhon Ratchasima, Thailand). After transportation in ice to a laboratory for 1 h, fish were prepared as fillets and shaped into 150 ± 25 g/piece (1.5 ± 0.5 cm thickness). The fillets were individually packed in commercial plastic bags and vacuum-sealed using a vacuum-packing machine (FVC-II, Furukawa MFG Co., Ltd., Chiba, Japan) with an extent of vacuum of 99.6%. Then, samples were divided into 6 groups and immersed in a SV 2447 vacuum cooker (Severin, Sauerland, Germany) for various temperatures and times as follows:

(1) 50 °C, 30 min (50-30)
(2) 50 °C, 45 min (50-45)
(3) 50 °C, 60 min (50-60)
(4) 60 °C, 30 min (60-30)
(5) 60 °C, 45 min (60-45)
(6) 60 °C, 60 min (60-60)

The control (C) of this study was tilapia fillet directly cooked with boiling water (100 °C) until the internal temperature of the meat reached 71 °C, at which point it was held for 5 min. During SV-cooking, temperature data logger series II (ThermaData-K, Chandler, AZ, USA) was used to monitor the temperature fluctuations. The thermocouple probe was inserted into each vacuum pouch with the tilapia fillet. After cooking, samples were then rapidly cooled down with tap water and subjected to further analysis.

### 2.2. Total Sulfhydryl (-SH) and Carbonyl Content

Myofibrillar proteins (MPs) were extracted using the method of Niu et al. [11]. Fish meat (5 g) was homogenized with 20 mmol/L Tris-HCl buffer containing 0.1 mol/L KCl (pH 7.5). The homogenate was centrifuged at 10,000× *g* for 20 min at 4 °C (Beckman Coulter, Avanti J-E Centrifuge, Palo Alto, Santa Clara, CA, USA) and the pellet was collected. After that, a 20 mmol/L Tris-HCl buffer containing 0.6 mol/L KCl (pH 7.0) was added to the pellet, homogenized and then incubated at 4 °C for 1 h. Samples were then centrifuged at 10,000× *g* for 15 min at 4 °C. The supernatant, which refers to the remaining MPs, was collected to analyze their total -SH content and carbonyl content as per the method of Ellman [12] and Chanarat et al. [13], respectively, and was expressed as nmol/mg protein.

### 2.3. Free Fatty Acid (FFA) Content

Lipids were extracted using a solvent mixture of chloroform methanol-distilled water (50:100:50, *v*/*v*) according to the method of Bligh and Dyer [14]. Then, lipid samples were determined by FFA content according to the method of Lowry and Tinsley [15]. Palmitic acid in isooctane (0–10 mM) was used to prepare a standard curve. FFA content was expressed as g FFA/100 g lipid.

### 2.4. Peroxide Value (PV) and Thiobarbituric Acid Reactive Substance (TBARS) Value

PV and TBARS values were measured using a UV-1601 spectrophotometer (Shimadzu) [16]. For PV, a standard curve was prepared using 0.5–2 ppm of cumene hydroperoxide and the results were expressed as mg hydroperoxide/kg sample. For the TBARS value, 0–2 ppm of malonaldehyde bis (dimethyl acetal) was used as standard and the results were expressed as a mg malonaldehyde (MDA)/kg sample.

### 2.5. Free Amino Acid Composition

The free amino acid composition of samples was determined as per the method of Minh-Thuy et al. [17]. Briefly, samples (25 g) were extracted using 6% (*v*/*v*) perchloric acid, and were subsequently neutralized and filtrated. After that, the filtrate was analyzed using an amino acid analysis system (Prominence, Shimadzu, Kyoto, Japan) equipped with a column (Shim-pack Amino-Li, 100 mm 9 6.0 mm i.d.; column temperature, 39.0 °C; Shimadzu) and pre-column (Shim-pack ISC-30/S0504 Li, 150 mm 9 4.0 mm i.d.; Shimadzu). A fluorescence detector (RF-10AXL; Shimadzu) was used to identify the free amino acids, which were reported in terms of a mg/100 g sample.

### 2.6. Volatile Compounds

Volatile compounds were analyzed by headspace solid phase microextraction gas chromatography mass spectrometry (HS-SPME GC-MS) following the method described by Pongsetkul et al. [18] with some modifications. Fish meat was ground using liquid nitrogen and a blender. Ground samples (5 g) were placed in a 20 mL headspace vial (Supelco, Bellefonte, PA, USA), then tightly capped with a PTFE septum and heated at 60 °C with an equilibrium time of 10 h. The SPME fiber (50/30 lm DVB/Carboxen™/PDMS StableFlex™) (Supelco, Bellefonte, PA, USA) was heated at 270 °C for 15 min before being exposed to the headspace. The 20 mL vials (Agilent Technologies, Palo Alto, CA, USA) containing the extracts were allowed to absorb into the SPME fiber at 60 °C for 1 h. The volatile compounds were then desorbed in the GC injector port for 15 min at 270 °C. After that, GC-MS analysis was performed in a HP 5890 series II gas chromatography (GC) coupled with HP 5972 mass-selective detector equipped with a splitless injector and coupled with a quadrupole mass detector (Hewlett Packard, Atlanta, GA, USA). The identified volatile compounds were presented in terms of abundance.

### 2.7. Adenosine Triphosphate (ATP)-Related Compounds

ATP-related compounds including adenosine triphosphate (ATP), adenosine diphosphate (ADP), adenosine monophosphate (AMP), inosine monophosphate (IMP), inosine (HxR) as well as hypoxanthine (Hx) were determined using HPLC as per the method of Kuda et al. [19]. Then, 2 g of sample were added with 10 mL of perchloric acid (10%, *v*/*v*), and then mixed and centrifuged at 8000× *g* for 5 min (4 °C) to collect the supernatant. The pellet was re-extracted in the same manner. All supernatants were combined and adjusted to 25 mL using 10% perchloric acid. The mixture was then neutralized with KOH, followed by centrifugation as described above. After that, the supernatants were filtered (Millex-LG 0.20 μm) and subjected to analysis using HPLC (Hitachi L2130, Hitachi koki Co., Ltd., Tokyo, Japan) equipped with column (Shodex GS-320 HQ, Showa Denko K.K., Tokyo, Japan). The condition included mobile phase: 200 mM NaH_2_PO_4_·2H_2_O, flow rate: 0.6 mL/min and temperature: 30 °C. A detector (Hitachi L7420, Hitachi koki Co., Ltd., Tokyo, Japan) was used and the absorbance of 260 nm was monitored. The results were expressed as μmol/g sample.

### 2.8. Sensory Evaluation

The nine-point hedonic scale was chosen to evaluate the consumer acceptability of SV-cooked tilapia processed by various conditions as per the method of Pongsetkul et al. [5]. Seventy-five untrained panelists (23 men and 52 women, 18–51 years old, living in Nakhon Ratchasima) who enjoy consuming tilapia were asked to assess the odor-, flavor- as well as overall-liking scores of the samples (1 = dislike extremely, 9 = like extremely). For sample preparation, SV-cooked fish were heated with a microwave oven (EMM20K18GW, Electrolux, Bangkok, Thailand) at full power (200 watts and a frequency of 2450 MHz) for 45 s before being served on a white paper plate, which was labeled with 3-digit random codes. Panelists were asked to evaluate the samples, take a sip of water after testing each sample and rate the score of each sensory attribute. The evaluation was done in individual sensory evaluation booths under fluorescent white light.

### 2.9. Statistical Analysis

All analyzes were conducted in triplicate (*n* = 3) in three lots of samples. Results are presented as means ± SD. Statistical analysis was performed using one-way analysis of variance (ANOVA). Mean comparison was carried out using Tukey’s test at the significance level of 95% (*p* < 0.05). SPSS package version 16.0 (SPSS for window, SPSS Inc., Chicago, IL, USA) was used for data analysis. Moreover, principal component analysis (PCA) was performed to assess the relationship between quality attributes related to flavor and odor characteristics and sensory scores of tilapia processed by various SV conditions.

## 3. Results and Discussion

### 3.1. Total -SH and Carbonyl Content

**Figure 1:**
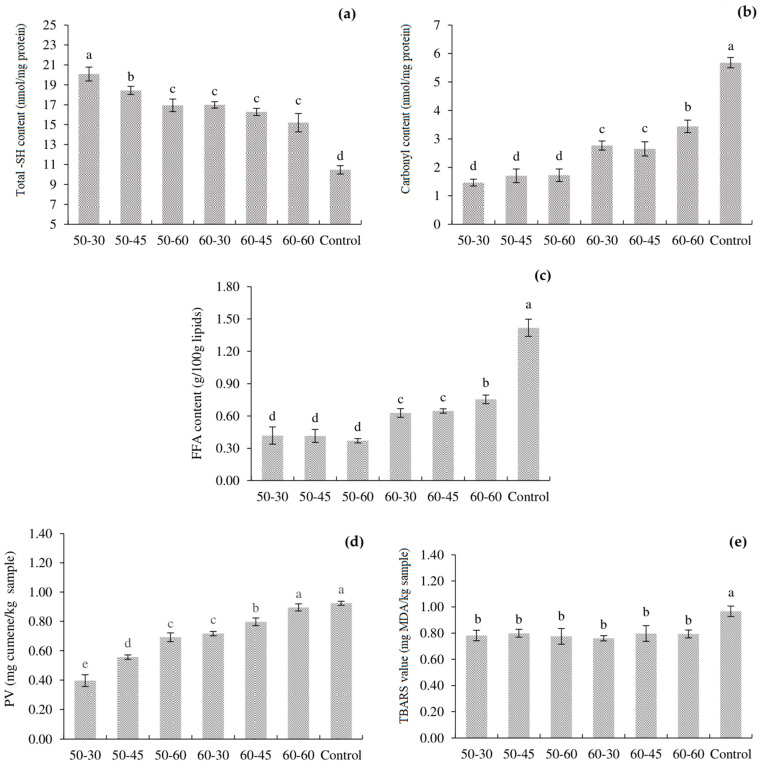
Total -SH content (**a**), carbonyl content (**b**), FFA content (**c**), PV (**d**) and TBARS value (**e**) of SV-cooked tilapia processed by various SV conditions. Different lowercase letters indicate significant differences (*p* < 0.05).

Figure 1a presents total -SH content of tilapia processed by various SV conditions. At 50 °C of SV cooking, the total -SH content significantly decreased when processed with a longer time (*p* < 0.05), whereas there was no significant difference between samples treated at 60 °C (*p* > 0.05). Among all samples, the lowest total -SH content was obtained in the control (*p* < 0.05), accounting for 10.46 nmol/mg protein. The decrease in the total -SH content might be associated with the unfolding or changing of MPs during heating, initiating the oxidation of hidden/surface SH groups [20]. The results were consistent with the carbonyl content, as depicted in Figure 1b. It was found that the control had the highest carbonyl content (5.68 nmol/mg protein), whereas various SV-cooked tilapia were found to be 1.47–3.34 nmol/mg protein. Generally, -SH and carbonyl contents are two vital indexes regarding the degree of protein oxidation. The formation of carbonyl groups has been noticed as one of the most outstanding modifications in oxidized proteins. The negative effect on meat quality governed during thermal processing might be caused by increasing reactive oxygen species (ROS) production and may have led to the higher conformational changes and denaturation/oxidation of the protein [21], which may promote a significant decrease in nutritional value in terms of availability of essential amino acids, i.e., lysine, and eating quality in terms of promoting some undesirable texture or flavor. In fact, oxidative or proteolytic reactions play a key role in the releasing of volatile compounds, which have a direct impact on the flavor and aroma of the meat [22]. From this experiment, cooked tilapia using the SV technique can lower the denaturation/oxidation of protein; thus, some nutritional value or characteristics of SV products may be improved and be better than traditional cooking.

### 3.2. FFA, PV and TBARS

The FFA content of traditional cooked tilapia (control) was 1.42 g/100 g lipids, whereas the FFA content of all SV-cooked tilapia was significantly lower, accounting for 0.37–0.76 g/100 g of total lipids (*p* < 0.05), as shown in Figure 1c. Among all SV-cooked samples, samples processed at 60 °C had higher FFA content, compared with samples processed at 50 °C at all processing times (*p* < 0.05), indicating lipid hydrolysis occurring at the higher level when applied with more severe SV conditions. Hydrolysis of glycerol-fatty acid esters can occur because of lipase and phospholipase being liberated from fish muscle, digestive organs as well as from microorganisms during heating, thus, resulting in the accumulation of FFA [7]. Generally, FFAs are susceptible to lipid oxidation. With increasing FFA during the process, lipid oxidation could proceed more rapidly and affect to cooked meat quality, particularly the odor/flavor to some extent.

PV and TBARS values, which represented the primary and secondary lipid oxidation products, of various SV-cooked tilapia, are presented in Figure 1d,e, respectively. Among all samples, 50-30 had the lowest PV, accounting for 0.40 mg cumene/kg sample, whereas the highest PV was obtained in the 60-60 sample and the control (0.90 and 0.92 mg cumene/kg sample, respectively). It was found that PV increased as cooking temperature or processing time increased (*p* < 0.05). Our results were in accordance with the results reported by Ismail et al. [9], who stated that higher and longer cooking temperatures and times of SV can tremendously increase the rate of lipid oxidation, as indicated by the increase in PV of cooked beef. In this experiment, the result suggested SV cooking of tilapia at 50–60 °C can accelerate the propagation stage of lipid oxidation but occur at the lower level if compared to traditional boiled fish. The highest TBARS value was also obtained in the control (0.97 mg MDA/kg sample), whereas all SV-cooked samples did not show significantly different TBARS values, which were in the range of 0.76–0.80 mg MDA/kg sample (*p* > 0.05). In fact, SV treatments are characterized by the absence of oxygen; thus, SV had been supposed to show lower lipid oxidation in comparison with traditional cooking. However, a close correlation between TBARS and sensory properties, particularly off-odor/flavor, has been noted in aquatic products [23]. The generally acceptable limit of TBARS is less than 2 mg MDA/kg sample, and higher than the limit is considered rancid [24]. In this experiment, the TBARS value of all samples was in the range of the limit; thus, it could be inferred that all samples may not owe their off-odor/rancidity to lipid oxidation.

### 3.3. Free Amino Acid Composition

The difference in free amino acid composition of SV-cooked tilapia processed by various SV conditions is shown in Table 1. All samples containing Gly, Lys, Glu, Ala and Pro were found to be dominant (>10 mg/100 g sample) and were similar with the dominant amino acids found in fresh tilapia, as reported by Cheng et al. [25] and Herath et al. [26]. Among all samples, 18 amino acids were detected at different amounts, suggesting that various processing temperatures and times affect the degradation rate of protein, resulting in the change of amino acid content in cooked meat. Generally, free amino acids contribute directly to taste, and participate indirectly in flavor development as well [27]. Glu and Asp are responsible for umami taste intensity, whereas Ala, Gly, Pro, Ser and Thr are related to the sweetness of cooked meat [9]. In this experiment, except for Pro, Ser and Thr, the contents of these umami and sweet amino acids were significantly improved when processed with higher temperatures and longer times for SV cooking. The highest total umami and sweet amino acids were obtained in the 60-45 and 60-60 samples, which were also slightly higher than the control, inferring that SV cooking may improve the flavor/taste of the final SV-cooked fish. This was in accordance with the higher flavor-liking scores found in these two SV-samples, compared with the control.

Hydrophobic amino acids, including Arg, His, Ile, Leu, Met, Phe, Trp and Val contribute to a bitter taste [9]. The results show that the content of total bitter amino acids was decreased when applied with the higher temperature and longer time of SV cooking. Similarly, total tasteless amino acids, including Cys, Lys and Tyr, were also decreased when applied to severe SV conditions. Although these tasteless amino acids seem not to be related with flavor/taste of samples, it can affect the nutritional value of the product. Among all samples, the control and 60-60 sample had the significant lowest levels of Lys, an essential amino acids (*p* < 0.05), indicating the loss of nutritional value when processed at more severe heating conditions.

### 3.4. Volatile Compounds

A total of 43 volatile compounds including aldehydes (8), ketones (8), alcohols (7), N-containing compounds (4), S-containing compounds (6) and others (3) were detected in various SV-cooked tilapia (Table 2). Overall, as expected, each sample varied in the proportion of volatile compounds, evidencing the influence of temperature and time on volatile profiles formed during SV cooking.

Normally, aldehydes and ketones are secondary products from lipid oxidation [28]. In depth, aldehydes are thermal degradation products of fats, whereas ketones are generated by microbial enzymatic actions on either lipids or amino acids, as well as by the Maillard reaction [29]. From the results, total abundance of aldehydes, i.e., 2,3-methylbutanal, hexanal, heptanal, 2-octenal, etc. of SV-cooked tilapia tends to decrease as the temperature and time for SV cooking increased. Furthermore, the total abundance of ketones, i.e., 2-butanone, 2-heptanone, 2-decanone, etc. tended to increase when processed with more severe conditions. The results stated that lipid oxidation took place during SV-cooking. However, because of the quite low temperature of SV cooking (50–60 °C), the aldehyde generated by thermal degradation of lipids may occur at a lower level in all samples. Dominguez et al. [22] reported that unsaturated acyl groups were slightly oxidized during SV cooking and led to the formation of ketones, alcohols, aldehydes and acids. Compared to aldehydes, ketones were governed and applied in more severe SV conditions. Among all ketones, acetoin was found to be dominant in all samples, accounting for 6.78–12.49%. Acetoin is responsible for the buttery, sweet, cream or classified as desirable oily note in meat products, as reported by Zhang et al. [30]. The diketones produced during the process are the products of the primary stage of the Maillard reaction, which can balance the flavor of meat [29]. Thus, the development of acetoin when processed with higher temperature and longer processing time of SV cooking may be associated with the improvement of some flavors in the final product. In general, ketones contain high odor threshold values [31]. Therefore, their aroma contributions might be minimal, even though their flavor notes are generally desirable.

Alcohol was found at moderate proportions in all SV-cooked tilapia (14.42–21.42%). Alcohol is also generated by lipid oxidation, and their thresholds are normally high, so they do not make great contributions to meat odor/flavor [28]. Among all alcohols, 1-Octen-3-ol was found to be the dominant alcohol in all samples, accounting for 10.12–17.06%. 1-Octen-3-ol, an unsaturated alcohol, is one of the main volatiles detected in fresh fish together with hexanal [29]. Thiansilakul et al. [32] reported that 1-octen-3-ol strongly contributed to the fishy or rancid off-odors in Asian seabass meat. It has a low detection threshold (1.5 μg/kg), which contributes to the mushroom aroma and can be eradicated during the heating process [28]. This description is related to our results, which found that 1-Octen-3-ol was found at lower amounts when applied with a higher temperature and longer SV cooking time. Moreover, the lower level of this compound, particularly when processed with more severe SV conditions, may improve the flavor/odor of cooked fish by lowering the fishy or rancid off odors occurring in fresh meat.

All SV-cooked tilapia with an N-containing compound as the major compound, accounts for more than 25% of all volatiles. Only four nitrogen-containing compounds, including trimethylamine, 2,6-dimethylpyrazine, trimethylpyrazine and 2-ethyl-3,5-dimethylpyrazine, were detected in all samples. It was found that trimethylamine was the most abundant volatile compound in SV-cooked tilapia, corresponding well with the reports by Li et al. [29] and Zhang et al. [28]. In this study, trimethylamine was found at the highest level in the control and the 50.30 sample, accounting for 30.25 and 28.42%, respectively. Among all SV-cooked samples, this compound was found at a lower amount when the temperature and time for cooking was increased, which was lower than the control. Trimethylamine has been reported in most fish species, contributing to the “fishy” aroma note with a low detection threshold. A high level of TMA lends an undesirable odor to seafood products [29]. The lower amount found in SV-cooked samples, compared with the control, may indicate that SV cooking could prohibit the development of undesirable odor/aroma compounds generated during heating better than the traditional method. The other three N-containing compounds found in the samples were pyrazine derivatives. Our results showed that the control contained higher pyrazine derivatives than the SV-cooked samples. Pyrazines were produced by the Maillard reaction and the thermal degradation reaction. These compounds were generally prevalent in thermally processed meat, particularly roasted meat, which is responsible for the nutty, earthy and fruity taste [18]. However, it accumulated in quite a low amount; thus, it could not be the main compound responsible for cooked/boiled fish.

Five S-containing compounds were detected. Among them all, methanethiol was found to be dominant (4.11–15.15%). The results indicated that this compound decreased when the temperature or time of SV-cooking increased. This corresponded to the study by Roldan et al. [33] and Zhang et al. [28] who reported that the formation of methanethiol in cooked tilapia and herring initially increased to a maximum, and then declined as thermal time increased, respectively. During heating, methanethiol, the breakdown product of aldehyde, is generated and plays a role as a precursor to forming numerous sulfur compounds such as dimethyldisulphide [33]. Zhang et al. [28] noted that the S-containing compound has very low odor thresholds and potent flavor characteristics, thus playing a key role in the aroma of cooked meat.

Moreover, limonene as well as two furans including 2-pentylfuran and 2-methylfuran were detected in the samples. However, in most cases, furans do not contribute a lot to the overall aroma in most fish species, particularly boiled fish. This was because the concentration found in boiled fish was below the detection threshold, as reported by Huang et al. [34].

### 3.5. ATP-Related Compounds

AMP and IMP were the dominant ATP-related compounds found in all SV cooked tilapia, as shown in Table 3. This corresponded to the studies by Llave et al. [3] and Miao et al. [35], who reported that SV-cooked tuna revealed a large accumulation in IMP content, compared with other ATP-related compounds. In fact, the degradation of ATP occurs rapidly after fish death. The formation of ATP breakdown products is temporarily accumulated as IMP occurs during the early stages of the storage or processing period [36]. It has been noted that IMP generally functions as a flavor enhancer and is responsible for the umami flavor of meaty foods [3]. Among all samples, 60-45 and 60-60 had the highest IMP content, accounting for 14.25–14.31 μmol/g sample. The highest accumulation of IMP may have been because of the desired heating temperatures and processing time achieved at the end of SV cooking, which may be further responsible for the desired flavor/taste of the product. The result found that the control had the highest HxR than all SV samples (*p* < 0.05). Among SVsamples, the increase in HxR was obtained when the temperature or time for SV cooking was increased, indicating the further degradation of IMP into HxR and finally Hx. However, there was no significant difference in the Hx content of all samples (*p* > 0.05). Overall, ATP-related compounds were found to be varied according to processing conditions. These compounds, along with other compounds, i.e., free amino acid, peptides, as well as volatile compounds, were plausibly involved in the desired flavor/taste of cooked fish.

### 3.6. Sensory Scores

The mean acceptability scores of the odor-, flavor- and overall-likeness of samples were evaluated (Table 3). The result found that there was no significant difference in odor-liking scores among all samples (*p* > 0.05), which had scores ranging from 6.58–6.88. In contrast, the improvement in the flavor-liking score of SV-cooked fish was obtained when the fish was processed with higher temperatures and longer times. Among all SV samples, 60-45 and 60-60 had the highest flavor-liking scores (*p* < 0.05), accounting for 7.88 and 7.72, respectively, which were significantly higher than the control (7.16). The higher IMP found in 60-45 and 60-60, which is one of the desirable flavor compounds, may lead to the higher umami taste or desirable flavor, compared with others. The overall-liking score seems to have strong positive correlations with flavor-liking scores, which found the highest scores in the 60-45 and 60-60 samples as well (*p* < 0.05). These two samples had overall-liking scores of 7.46 and 7.62, which were significantly higher than the control (6.56). On the nine-point hedonic scale, a mean liking score of 7.00 or higher is generally claimed as having a highly acceptable sensory quality [37]. Interestingly, it can be observed that the control had an lower overall-liking score than the SV-cooked samples (*p* < 0.05), which was lower than 7.00. This may be due to the texture characteristics, which were note focused on/mentioned in this study. In conclusion, the results clearly demonstrated that SV-cooked tilapia, particularly cooked at 60 °C for 45 and 60 min, provided a prime or desirable flavor better than traditional/directly cooked boiled fish, thus resulting in better overall acceptability of the product.

### 3.7. PCA

**Figure 2:**
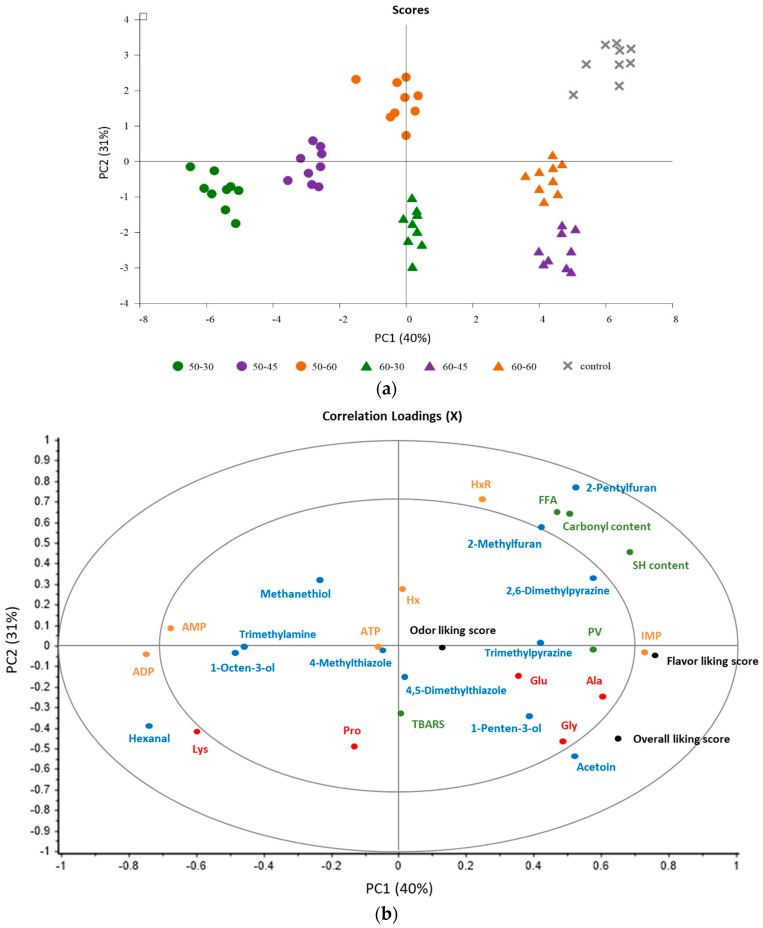
PCA score plots (**a**) and correlation loading (protein/lipid oxidation products (green color), some free amino acids (red color), some volatiles (blue color), ATP-related compounds (orange color) and sensory scores (black color)) plots (**b**) of SV-cooked tilapia processed by various SV conditions.

The score plot and correlation loading expressed the relationship between some crucial meat characteristics/compounds and the sensory scores of SV-cooked samples, as shown in Figure 2a,b, respectively. Approximately 71% of the total variation was counted to explain this relationship. From Figure 2b, all variables located in the outer circle region exhibited a significant correlation with the seven experimental groups with a variance greater than 70%. It was found that the 60-45 and 60-60 samples, which were located at the lower part of the right side, were positively correlated with IMP, acetoin as well as flavor- and overall liking scores. For the control, which were located at the upper part of the right side, a positive correlation was found with HxR, FFA, -SH and carbonyl content as well as 2-pentylfuran, whereas the 50-30 and 50-45 samples, located at the lower part of the left side, were significantly correlated with ADP, Lys and hexanal. Conversely, the 50-60 and 60-30 samples appeared to show little correlation with other quality parameters.

Based on the PCA results, it helps to confirm that the flavor-liking score was positively correlated with the overall-liking score of SV-cooked fish. The highest flavor-liking score of the 60-45 and 60-60 samples might be influenced by higher IMP and acetoin. The results also suggested that IMP and acetoin are crucial flavor enhancers for cooked tilapia. The positive correlation between FFA, -SH and carbonyl content along with the control sample indicated that protein and lipid oxidation mainly occurred when the fish was processed by the traditional method, compared with SV cooking at 50–60 °C for up to 1 h. These oxidation products were also significantly correlated with the generation of 2-pentylfuran. Even furan does not contribute a lot to the overall aroma in boiled fish, as stated by Huang et al. [34]. However, 2-pentylfuran is known to be mainly responsible for the undesirable reversion flavor of other foods, i.e., Dezhou braised chicken [38], soybean oil [39], apple juice [40], etc. The higher rate of oxidation of traditional cooking, therefore, may influence the lower flavor-likeness of the control sample. Overall, this study confirmed that the SV-cooking technique can improve the meat quality of cooked fish, in terms of flavor/taste characteristics, particularly when processed at 60 °C for 45 and 60 min.

## 4. Conclusions

SV cooking at different temperatures and times is responsible for a significant change to non-volatile and volatile compounds, which contribute to the flavor/taste characteristics of tilapia meat. In this experiment, SV cooked at 60 °C for 45 and 60 min (60-45 and 60-60) possessed higher inosine monophosphate (IMP), acetoin, explaining the higher umami or desirable flavor/taste for these treatments. These two optimal SV conditions also lead to a significantly higher flavor-liking score in the consumer assessment, leading to the higher overall-liking score, compared to traditional cooked tilapia. The finding confirmed that the SV technique, at the optimal condition, can improve flavor/taste characteristics of cooked tilapia to some extent.

## Figures and Tables

**Table 1:** Free amino acid composition of SV-cooked tilapia processed by various SV conditions (mg/100 g).

Category	Name	50-30	50-45	50-60	60-30	60-45	60-60	Control
Umami	Aspartic acid (Asp)	5.45 ± 1.01 ^b^	7.78 ± 1.22 ^a^	8.99 ± 1.66 ^a^	9.33 ± 2.02 ^a^	7.12 ± 1.63 ^ab^	8.59 ± 1.67 ^a^	7.14 ± 1.44 ^a^
	Glutamic acid (Glu)	30.66 ± 2.99 ^b^	30.33 ± 2.05 ^b^	36.06 ± 3.66 ^a^	33.21 ± 3.41 ^ab^	36.77 ± 2.06 ^a^	38.02 ± 1.99 ^a^	34.34 ± 3.06 ^ab^
	Total	36.11	38.11	45.05	42.54	43.89	46.61	41.48
Sweet	Alanine (Ala)	25.51 ± 2.68 ^b^	25.08 ± 4.55 ^b^	23.23 ± 2.16 ^b^	27.12 ± 2.44 ^b^	26.66 ± 2.19 ^b^	33.99 ± 3.03 ^a^	23.03 ± 4.11 ^b^
	Glycine (Gly)	53.22 ± 4.15 ^ab^	46.77 ± 8.02 ^b^	50.11 ± 7.81 ^ab^	54.54 ± 5.55 ^ab^	66.02 ± 8.08 ^a^	60.09 ± 9.63 ^ab^	56.66 ± 8.95 ^ab^
	Proline (Pro)	10.03 ± 1.02	12.15 ± 1.45	12.66 ± 2.02	10.99 ± 2.33	11.52 ± 2.05	13.13 ± 2.01	13.06 ± 2.12
	Serine (Ser)	0.89 ± 0.21	1.03 ± 0.16	0.88 ± 0.17	0.89 ± 0.20	0.92 ± 0.21	0.90 ± 0.19	0.97 ± 0.23
	Threonine (Thr)	2.56 ± 0.29	2.33 ± 0.18	2.54 ± 0.20	2.61 ± 0.25	2.30 ± 0.20	2.45 ± 0.16	2.22 ± 0.21
	Total	92.21	87.36	89.42	96.15	107.42	97.43	95.28
Bitter	Arginine (Arg)	2.33 ± 0.26 ^b^	2.65 ± 0.33 ^ab^	3.03 ± 0.34 ^a^	2.33 ± 0.29 ^b^	1.47 ± 0.32 ^c^	1.35 ± 0.32 ^c^	1.33 ± 0.35 ^c^
	Histidine (His)	4.65 ± 0.23 ^a^	4.44 ± 0.19 ^a^	4.54 ± 0.15 ^a^	4.71 ± 0.15 ^a^	4.11 ± 0.20 ^b^	3.72 ± 0.29 ^b^	4.04 ± 0.17 ^b^
	Isoleucine (Ile)	0.55 ± 0.09	0.50 ± 0.10	0.47 ± 0.07	0.47 ± 0.07	0.42 ± 0.08	0.42 ± 0.06	0.49 ± 0.10
	Leucine (Leu)	5.62 ± 0.32 ^a^	5.23 ± 0.26 ^a^	5.05 ± 0.26 ^a^	3.99 ± 0.19 ^b^	3.90 ± 0.21 ^b^	4.02 ± 0.15 ^b^	3.62 ± 0.15 ^c^
	Methionine (Met)	0.36 ± 0.08	0.35 ± 0.09	0.30 ± 0.06	0.34 ± 0.09	0.36 ± 0.07	0.36 ± 0.05	0.33 ± 0.08
	Phenylalanine (Phe)	0.29 ± 0.05	0.30 ± 0.06	0.34 ± 0.06	0.29 ± 0.05	0.33 ± 0.06	0.32 ± 0.06	0.33 ± 0.05
	Tryptophan (Trp)	0.08 ± 0.02	0.09 ± 0.02	0.09 ± 0.03	0.12 ± 0.04	0.09 ± 0.02	0.09 ± 0.01	0.10 ± 0.03
	Valine (Val)	0.13 ± 0.03 ^a^	0.16 ± 0.02 ^a^	0.16 ± 0.03 ^a^	0.09 ± 0.02 ^ab^	0.07 ± 0.02 ^b^	0.09 ± 0.02 ^ab^	0.06 ± 0.02 ^b^
	Total	14.01	13.72	13.98	12.34	10.75	10.37	10.30
Tasteless	Cysteine (Cys)	ND	ND	ND	ND	ND	0.02 ± 0.00	0.03 ± 0.01
	Lysine (Lys)	32.32 ± 2.42 ^a^	30.16 ± 2.15 ^a^	30.50 ± 3.50 ^a^	26.24 ± 2.22 ^b^	22.01 ± 2.04 ^c^	20.99 ± 2.22 ^cd^	17.16 ± 3.08 ^d^
	Tyrosine (Tyr)	3.05 ± 0.19 ^a^	2.88 ± 0.13 ^a^	3.10 ± 0.16 ^a^	3.04 ± 0.11 ^a^	3.02 ± 0.09 ^a^	2.96 ± 0.12 ^a^	2.56 ± 0.15 ^b^
	Total	35.37	33.04	33.60	29.28	25.03	23.95	19.72
Total amino acids		177.70	172.23	182.05	180.31	187.09	178.36	166.78

Data appeared as mean ± SD from triplicate determinations. ND = Not detected. Different lowercase superscripts in the same row indicate the significant difference (*p* < 0.05).

**Table 2:** Volatile profile SV-cooked tilapia processed by various SV conditions.

Volatile Compounds ^1^	Aroma Description ^2^	RI ^3^	50-30	50-45	50-60	60-30	60-45	60-60	Control
Aldehydes									
Butanal	Pungent, fatty	891	0.23	0.22	0.15	0.28	0.13	0.21	0.30
2,3-Methylbutanal	Nutty, chocolate	902	0.55	1.08	0.26	0.35	0.24	0.16	0.21
Hexanal	Green, grassy	1050	5.42	3.66	3.02	1.67	1.62	1.04	0.67
Heptanal	Green, fatty	1180	1.05	1.23	1.22	1.02	1.43	1.33	1.45
2,4-Heptadienal	Fatty taste, fishy smell	1128	0.66	0.42	0.51	0.25	0.22	0.12	0.22
Nonanal	Green, citrusy	1374	0.44	0.40	0.36	0.36	0.21	0.33	0.29
2-Octenal	Oily, nutty	977	1.03	1.28	1.20	0.41	0.23	0.35	0.61
Benzaldehyde	Pleasant almond, nutty	1563	0.23	0.16	0.44	0.69	1.02	1.55	2.06
Total aldehydes			9.61	8.45	7.16	5.03	5.10	5.09	5.81
Ketones									
2-Butanone	Ethereal, acetone-like	902	0.66	1.25	1.03	1.22	0.39	0.23	0.28
2-Heptanone	Green taste, citrus flavor	1145	0.31	0.26	0.55	0.34	0.40	1.03	2.25
5-Hepten-2-one, 6-methyl-	Fatty, metallic	1025	0.23	0.19	0.22	0.24	0.20	ND	ND
2-Nonanone	Oily, plastic	1388	0.14	0.33	0.36	ND	ND	ND	ND
3-Octanone	Mushroom	983	0.21	0.17	0.17	0.12	0.22	0.14	0.15
2-Decanone	Floral, orange, fatty	1492	1.02	1.37	1.34	1.21	0.44	0.38	0.46
2-Undecanone	Orange, fatty	1201	0.34	0.41	0.55	0.21	0.12	0.16	0.12
Acetoin	Buttery, sweet, cream	1296	7.03	6.78	8.88	10.08	10.15	12.49	8.16
Total ketones			9.94	10.76	13.10	13.42	11.92	14.43	11.42
Alcohols									
1-Penten-3-ol	Balsam	882	0.98	0.66	2.02	1.64	2.69	2.70	1.08
1-Hexanol	Green, herbal	1360	0.66	2.01	1.69	1.33	1.45	1.92	0.54
1-Hexanol, 2-ethyl-	Citrus	960	0.31	0.26	0.75	0.38	0.44	0.56	1.02
1-Heptanol	Oily, balsam	1114	0.21	0.66	0.82	0.43	0.18	0.21	0.22
2-Nonen-1-ol	Cucumber, oily	1206	ND	0.44	0.67	ND	ND	ND	ND
1-Octen-3-ol	Mushroom, earthy	1438	16.55	17.06	14.05	14.62	14.21	13.22	10.12
1-Octanol	Mushroom, waxy	1556	ND	0.33	1.01	0.67	1.35	1.02	1.44
Total alcohols			18.71	21.42	21.01	19.07	20.32	19.63	14.42
N-containing compounds									
Trimethylamine	Ammonia, fishy, pungent	587	28.42	28.21	24.68	25.05	23.95	16.15	30.25
2,6-Dimethylpyrazine	Nutty, roasted, popcorn	1309	4.06	4.55	3.02	2.55	4.45	5.03	7.02
Trimethylpyrazine	Nutty, roasted, earthy	1401	1.42	1.03	1.48	1.65	1.78	4.26	3.11
2-Ethyl-3,5-dimethylpyrazine	Nutty, roasted, burnt	1438	ND	ND	ND	ND	0.11	ND	0.31
Total N-containing compounds			33.90	33.79	29.18	29.25	30.29	25.44	40.69
S-containing compounds									
Methanethiol	Pungent, sulphury	622	13.22	12.23	12.66	15.15	13.02	10.92	4.11
4-Methylthiazole	Cooked meat	1298	1.02	2.22	2.09	1.51	2.67	2.33	1.02
2-Methylthiazoline	Cooked meat	1436	ND	ND	ND	ND	0.21	0.33	0.09
4,5-Dimethylthiazole	Cooked meat	1380	3.45	3.05	3.03	2.99	4.87	3.05	1.05
Dimethyl trisulphide	Meaty, onion	970	0.41	0.63	0.95	0.66	0.60	1.12	0.65
Dimethyl tetrasulphide	Meaty	1015	0.25	0.65	0.99	0.56	0.24	0.32	0.41
Total S-containing compounds			18.35	18.78	19.72	20.87	21.61	18.07	7.33
Others									
Limonene	Citrus, orange	1190	1.73	2.02	1.44	0.54	0.50	0.56	ND
2-Pentylfuran	fruity	1229	4.03	2.65	4.44	5.27	6.08	6.22	10.15
2-Methylfuran	fruity	1027	3.52	1.86	3.65	5.43	4.08	10.02	10.11
Total others			9.28	6.53	9.53	11.24	10.66	16.80	20.26
Total peak abundance			99.79	99.73	99.70	98.88	99.90	99.46	99.93

^1^ Expressed as percentage of relative peak area of total peak. ND: not detectable. ^2^ Aroma description were from literatures [28,30,31]. ^3^ Retention index were compared with mass spectral database (NIST and Wiley libraries) and by comparison of their RI with those available in the literatures [28,31,33].

**Table 3:** ATP-related compounds and sensory scores of SV-cooked tilapia processed by various SV conditions.

Parameters	50-30	50-45	50-60	60-30	60-45	60-60	Control
ATP-related compounds (μmol/g sample)							
ATP	0.25 ± 0.04	0.21 ± 0.03	0.19 ± 0.06	0.25 ± 0.03	0.24 ± 0.05	0.22 ± 0.04	0.25 ± 0.03
ADP	0.87 ± 0.05 ^a^	0.86 ± 0.03 ^a^	0.64 ± 0.03 ^b^	0.85 ± 0.02 ^a^	0.55 ± 0.04 ^c^	0.56 ± 0.03 ^c^	0.48 ± 0.05 ^c^
AMP	5.30 ± 0.12 ^a^	5.43 ± 0.09 ^a^	4.29 ± 0.10 ^c^	5.02 ± 0.12 ^b^	4.18 ± 0.27 ^c^	4.26 ± 0.22 ^c^	4.07 ± 0.06 ^c^
IMP	12.59 ± 0.85 ^c^	13.09 ± 0.69 ^bc^	13.17 ± 0.21 ^b^	13.14 ± 0.35 ^b^	14.31 ± 0.47 ^a^	14.25 ± 0.70 ^a^	13.30 ± 0.38 ^b^
HxR	0.88 ± 0.03 ^b^	0.91 ± 0.04 ^b^	0.91 ± 0.05 ^b^	0.90 ± 0.06 ^b^	0.94 ± 0.07 ^b^	0.92 ± 0.04 ^b^	2.89 ± 0.06 ^a^
Hx	0.27 ± 0.05	0.28 ± 0.04	0.25 ± 0.02	0.25 ± 0.03	0.27 ± 0.03	0.25 ± 0.03	0.26 ± 0.03
Sensory scores							
Odor liking score	6.58 ± 0.43	6.82 ± 0.28	6.86 ± 0.29	6.90 ± 0.31	6.76 ± 0.27	6.88 ± 0.32	6.78 ± 0.29
Flavor liking score	6.20 ± 0.25 ^d^	6.52 ± 0.34 ^cd^	6.60 ± 0.23 ^cd^	6.70 ± 0.22 ^c^	7.88 ± 0.31 ^a^	7.72 ± 0.25 ^a^	7.16 ± 0.25 ^b^
Overall liking score	6.36 ± 0.31 ^c^	6.74 ± 0.28 ^c^	6.60 ± 0.30 ^c^	7.04 ± 0.22 ^b^	7.46 ± 0.21 ^a^	7.62 ± 0.37 ^a^	6.56 ± 0.24 ^c^

Mean ± SD from triplicate determinations. Different lowercase superscripts in the same row indicate the significant difference (*p* < 0.05).

## Data Availability

The data presented in this study are available on request from the corresponding author (J.P.).
